# Recurrent *NFIA* K125E substitution represents a loss‐of‐function allele: Sensitive in vitro and in vivo assays for nontruncating alleles

**DOI:** 10.1002/ajmg.a.62226

**Published:** 2021-05-11

**Authors:** Tomoko Uehara, Rikako Sanuki, Yurie Ogura, Atsushi Yokoyama, Takeshi Yoshida, Hiroshi Futagawa, Hiroshi Yoshihashi, Mamiko Yamada, Hisato Suzuki, Toshiki Takenouchi, Kohei Matsubara, Hiromi Hirata, Kenjiro Kosaki, Toshiyuki Takano‐Shimizu

**Affiliations:** ^1^ Center for Medical Genetics Keio University School of Medicine Tokyo Japan; ^2^ Advanced Insect Research Promotion Center Kyoto Institute of Technology Kyoto Japan; ^3^ Department of Chemistry and Biological Science College of Science and Engineering, Aoyama Gakuin University Sagamihara Kanagawa Japan; ^4^ Department of Pediatrics Kyoto University Graduate School of Medicine Tokyo Japan; ^5^ Department of Genetics Tokyo Metropolitan Children's Medical Center Tokyo Japan; ^6^ Department of Pediatrics Keio University Hospital Tokyo Japan

**Keywords:** corpus callosum anomaly, loss‐of‐function, model organisms, *NFIA*

## Abstract

*Nuclear factor I A* (*NFIA*) is a transcription factor that belongs to the NFI family. Truncating variants or intragenic deletion of the *NFIA* gene are known to cause the human neurodevelopmental disorder known as *NFIA*‐related disorder, but no patient heterozygous for a missense mutation has been reported. Here, we document two unrelated patients with typical phenotypic features of the *NFIA*‐related disorder who shared a missense variant p.Lys125Glu (K125E) in the *NFIA* gene. Patient 1 was a 6‐year‐old female with global developmental delay, corpus callosum anomaly, macrocephaly, and dysmorphic facial features. Patient 2 was a 14‐month‐old male with corpus callosum anomaly and macrocephaly. By using Drosophila and zebrafish models, we functionally evaluated the effect of the K125E substitution. Ectopic expression of wild‐type human *NFIA* in *Drosophila* caused developmental defects such as eye malformation and premature death, while that of human *NFIA* K125E variant allele did not. *nfia*‐deficient zebrafish embryos showed defects of midline‐crossing axons in the midbrain/hindbrain boundary. This impairment of commissural neurons was rescued by expression of wild‐type human *NFIA*, but not by that of mutant variant harboring K125E substitution. In accordance with these in vivo functional analyses, we showed that the K125E mutation impaired the transcriptional regulation of *HES1* promoter in cultured cells. Taken together, we concluded that the K125E variant in the *NFIA* gene is a loss‐of‐function mutation.

## 1 INTRODUCTION

The prevalence of intragenic deletions in patients with aplasia or hypoplasia of the corpus callosum and developmental delay suggests that haploinsufficiency of the *Nuclear factor I A* (*NFIA*) gene is a primary cause of chromosome 1p32‐p31 deletion syndrome or brain malformations with or without urinary tract defects (MIM 613735) (Bayat et al., 2017; Hollenbeck et al., 2017; Mikhail et al., 2011; Nyboe et al., 2015; Rao et al., 2014). Identification of frameshift and nonsense mutations in *NFIA* further supports this notion (Negishi et al., 2015; Revah‐Politi et al., 2017; Zhang et al., 2020). Indeed, the mouse ortholog of this conserved transcription factor, *Nfia*, is required for differentiation and maturation of astrocyte and oligodendrocyte and its loss results in the aplasia/hypoplasia of corpus callosum and urinary tract defects (das Neves et al., 1999; Lu et al., 2007).

Despite growing recognition of the impact of *NFIA* haploinsufficiency on the neurodevelopmental disorder, there is no patient heterozygous for a pathogenic missense variant to date (but see also Zenker et al., 2019 for three candidate pathogenic variants). Here, we report the same de novo missense mutation K125E in the *NFIA* gene in two unrelated patients. By using *Drosophila* and zebrafish models (Suzuki et al., 2019; Uehara et al., 2020) as well as cell culture system, we unambiguously demonstrated that this K125E missense variant represents a loss‐of‐function pathogenic allele. Presently reported in vivo assays will be useful for functional evaluation of other missense variants of *NFIA*.

## 2 CLINICAL REPORT

**FIGURE 1:**
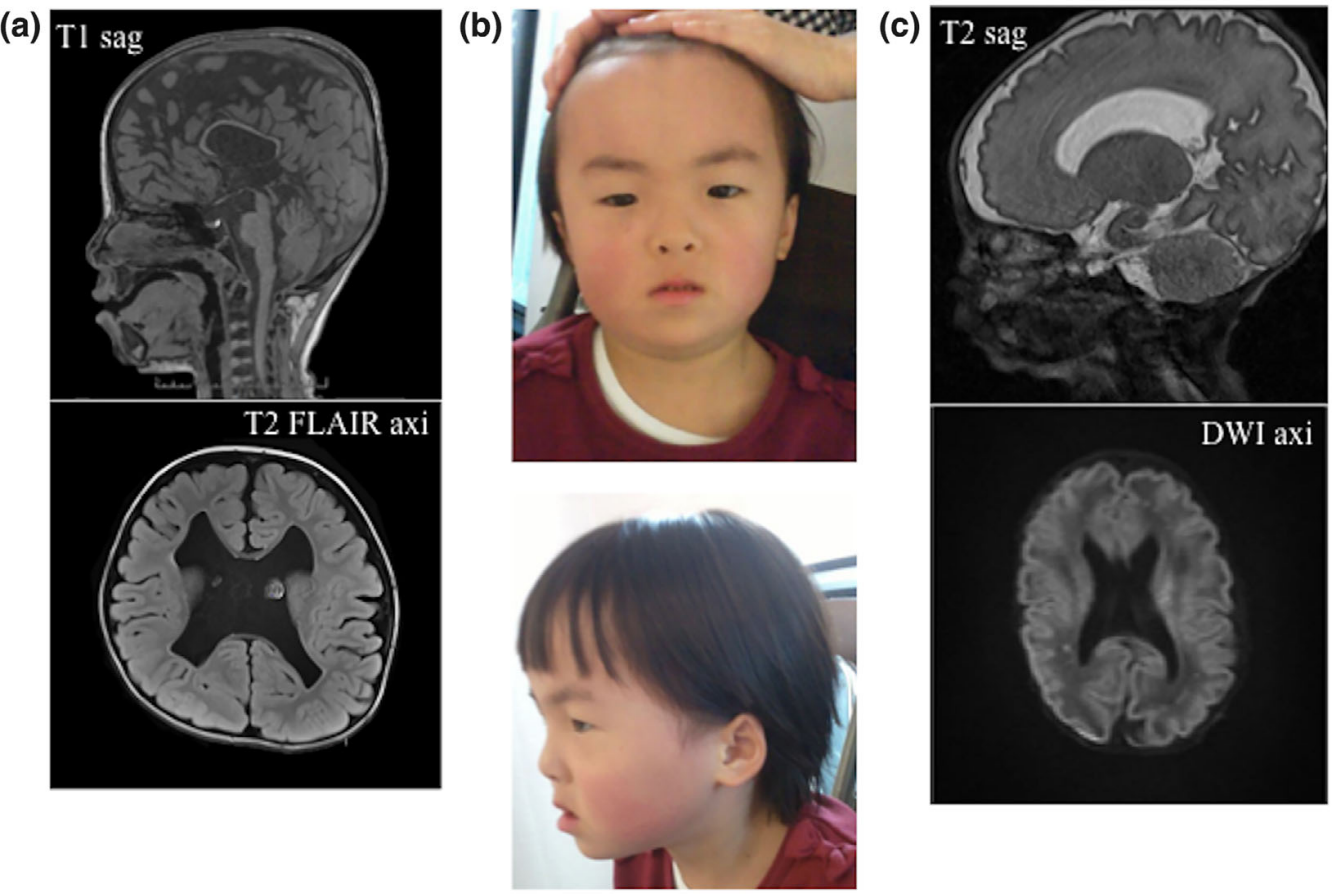
Clinical characteristics of two patients with the same *NFIA* variant. (a) Results of head MRI of Patient 1 at 5 years of age. The picture above shows sagittal T1‐weighted image. The picture below shows axial T2‐weighted fluid‐attenuated inversion recovery image. Note thin corpus callosum, cyst of septi pellucidi, ventricular wall irregularity, and decreased white matter volume. (b) Pictures of Patient 1 at 6 years of age. Note the high hairline, small eyes, anteverted nares, a depressed nasal bridge, a broad columella, and a thin upper‐lip. (c) Results of head MRI of Patient 2 at 1 month of age. The picture above shows sagittal‐T2‐weighted image. The picture below shows axial diffusion‐weighted image. Note polycerebral gyrus at parasylvius fissures, cortical dysplasia of bilateral cerebral hemisphere, partial myelination delay, and hypoplasia of corpus callosum [Color figure can be viewed at wileyonlinelibrary.com]

Patient 1 was a 6‐year‐old female who was the first child of healthy and nonconsanguineous Japanese parents. She was born after an uncomplicated pregnancy at 35 weeks and 4 days of gestation. Her weight at birth was 2142 g (−0.5 *SD*), length was 43 cm (−1.2 *SD*), and head circumstance was 33 cm (+0.8 *SD*). After birth, she showed tachycardia. Head ultrasound showed ventricular enlargement and intraventricular hemorrhage. She had been in neonatal intensive care unit since 34‐day‐old. After discharge, she attended her hospital regularly due to cerebral palsy and global developmental delay. At 2 years of age, she underwent an operation for exotropia. She had congenital hearing loss and wore hearing aids at 5 years of age. Head magnetic resonance imaging (MRI) at 5 years of age showed thin corpus callosum, cyst of septi pellucidi, ventricular wall irregularity, and periventricular leukomalacia (Figure 1a). She showed distinctive dysmorphic features with high hairline, small eyes, anteverted nares, a depressed nasal bridge, a broad columella, a thin upper‐lip, and high‐arched palate (Figure 1b). Her developmental milestones were delayed. She started to walk at the age of 3 years. She also stated to speak her words at the age of 3 years and spoke only few words at the age of 6 years. Her developmental quotient as assessed using the WISC‐IV test was 23. Her physical growth was also delayed. At 6 years of age, her weight was 15.9 kg (−1.3 *SD*), height was 102.4 cm (−2.4 *SD*), and head circumference was 54.5 cm (+2.4 *SD*). She had no urogenital anomalies.

Patient 2 was a 14‐month‐old boy who was born at 34 weeks gestation. He had been diagnosed at 34 weeks gestation with a head enlargement. His weight at birth was 2635 g (+2.1 *SD*), length was 47.5 cm (+1.6 *SD*), and head circumference was 35.3 cm (+3.0 *SD*). After birth, a head MRI showed polycerebral gyrus at parasylvius fissures, cortical dysplasia of bilateral cerebral hemisphere, partial myelination delay, and hypoplasia of corpus callosum (Figure 1c). He had mild congenital hearing impairment. An electrocardiogram and a renal echogram showed no anomalies. He showed distinctive dysmorphic features with high hairline, thick eyebrow, short nose, anteverted nares, long philtrum, thin upper‐lip vermilion, and a retrognathia. His developmental milestones were delayed; he gained head control and rolling over at 8 months of age, sat without support at 11 months of age, and slithering at 1 year of age. He was able to stand with support at 1 year of age. At 1 year and 1 month of age, his weight was 10.42 kg (+0.3 *SD*), height was 81.0 cm (+1.9 *SD*), and head circumference was 52.0 cm (+3.9 *SD*).

## 3 METHODS

### 3.1 Mutation analysis

Approval from the local institutional review board and informed consent from the patients' parents were obtained prior to the molecular studies. Whole exome sequencing using a SureSelect XT Human All Exon V6 Panel (Agilent Technology, Santa Clara, California) on a HiSeq platform (Illumina, San Diego, California) was performed for Patient 1 and her parents. Medical exome sequencing using the TruSight One Sequencing Panel (Illumina) on a MiSeq platform (Illumina) was performed for Patient 2 and his parents. We confirmed their results by performing Sanger sequencing with the following primers: NFIA_sense, 5′‐AAA ACC AGA GGT CAA GCA GAA G‐3′, and NFIA_antisense, 5′‐ATT CTC ACC ATC GCA CTT ACC T‐3′.

### 3.2 Functional assays in *Drosophila*

We PCR‐amplified the *NFIA* open reading frame sequence from a human cDNA clone (Kazusa DNA Research Institute, Chiba, Japan, ORK00836) and subcloned it into a modified pENTR221 vector. The K125E mutation was introduced into the subclone by site‐directed mutagenesis. Both subcloned fragments were verified by sequencing, and transferred into a destination vector pUASg‐attB (Bischof et al., 2013) via the LR reaction (Invitrogen). The plasmid DNA was injected into embryos carrying the attP40 landing site for phiC31 integrase‐mediated transformation (*y* ^*1*^ *v* ^*1*^ *P{y[+t7.7]=nos‐phiC31\int.NLS}X; P{y[+t7.7]=CaryP} attP40*, Bloomington Drosophila Stock Center, #25709). The wildtype and mutant *NFIA* transgenes were expressed via the *GAL4/UAS* system. The *GAL4* drivers used in this study were *nSyb‐GAL4* (*y[1] w[1118]; P{y[+t7.7] w[+mC]=nSyb‐GAL4.P}attP2*, Bloomington Drosophila Stock Center, BDSC# 51941) for pan‐neuronal expression, *GMR‐GAL4* (*w; P{w[+mC]=GAL4‐ninaE.GMR}12*, KYOTO Stock Center, DGRC# 106207) for expression in the retina, and *ey‐GAL4* (w; P{w[+m*]=GAL4‐ey.H}3‐8, KYOTO Stock Center, DGRC# 108283) for expression in the eye‐antenna disc.

### 3.3 Functional assays in zebrafish

Zebrafish (*Danio rerio*) were reared and maintained under a 14 h light and 10 h dark photoperiod according to the standard protocol. Zebrafish carrying the *nfia* Q232X nonsense mutant allele (*nfia* ^*sa16768*^) was obtained from Zebrafish International Resource Center and was used for rescue experiments with wild‐type and K125E mRNA. *nfia* ^*sa16768*^ was previously generated by a targeting induced local lesions in genomes project (Kettleborough et al., 2013). For rescue experiments, wild‐type human *NFIA* coding sequence was generated by a DNA synthesis service (Fasmac, Japan) and subcloned into an expression vector pCS2+. The K125E point mutation was introduced into human *NFIA* by the QuickChange method (Agilent Technologies) using following two primers; 5′‐GCTGCACAAACTCTTTAAGCATTTCTTGGGGATGGTATCTAATG‐3′ and 5′‐CATTAGATACCATCCCCAAGAAATGCTTAAAGAGTTTGTGCAGC‐3′. These constructs were used to generate wild‐type and mutant *NFIA* mRNA using the mMESSAGE mMACHINE SP6 Kit (Thermo Fisher Scientific) according to the manufacture's protocol. The human *NFIA* mRNAs (100 pg) were injected into 1–2‐cell stage zebrafish embryos produced by crossing *nfia* heterozygous mutant carrier fish. Embryos were fixed at 72 h postfertilization (hpf) and subjected to immunolabeling using anti‐acetylated α‐tubulin (clone 6‐11B‐1; Sigma), HRP‐conjugated anti‐mouse IgG (Invitrogen), and ImmPACT DAB Substrate (Vector Laboratories). For genotyping of immunolabeled embryos, the region surrounding the Q232X mutation site was amplified by genomic PCR using following two primers; 5′‐CTGTATTCTGTCATGTTCATTCAGATAACAGTC‐3′ and 5′‐GCTCAATGATGTCCCAAAAGGAAG‐3′. PCR products were digested with *Fai* I restriction enzyme (SibEnzyme, Russia) and separated by 15% polyacrylamide gel electrophoresis. This zebrafish study was approved by Animal Care and Use Committee of Aoyama Gakuin University (A9/2020) and carried out according to the Aoyama Gakuin University Animal Care and Use Guideline.

### 3.4 Cell culture

293T cells (RCB2202, provided by RIKEN BRC through the National Bio‐Resource Project of MEXT, Japan) were grown in Dulbecco's modified Eagle's medium (044–29765, Fujifilm Wako Pure Chemical, Osaka, Japan), supplemented with 10% fetal bovine serum (Biowest, Nuaillé, France) and a penicillin–streptomycin solution (Nacalai Tesque, Kyoto, Japan), and cultured at 37°C and 5% CO2 condition.

### 3.5 NanoLuc reporter assay

The human 0.6‐kb (−602 to +44) *GFAP* and 1.2‐kb (−1039 to +135) *HES1* promoter regions were amplified by PCR from human genome and were subcloned into the reporter vector *pNL2*.2 (Promega Corp., Madison, Wisconsin). The *pNL2.2* reporter constructs were cotransfected with *pCAGGS‐Luc2*, and *pCAGGS‐NFIA‐3xFlag* plasmids into 293T cells using the calcium phosphate‐mediated method. Two days later, the cells were lysed in passive lysis buffer (Promega Corp.), and the luciferase and NanoLuc activities were measured using the Nano‐Glo Dual‐Luciferase reporter assay system (Promega Corp.) according to the manufacturer's instructions.

## 4 RESULTS

### 4.1 Variants identified in two patients

**FIGURE 2:**
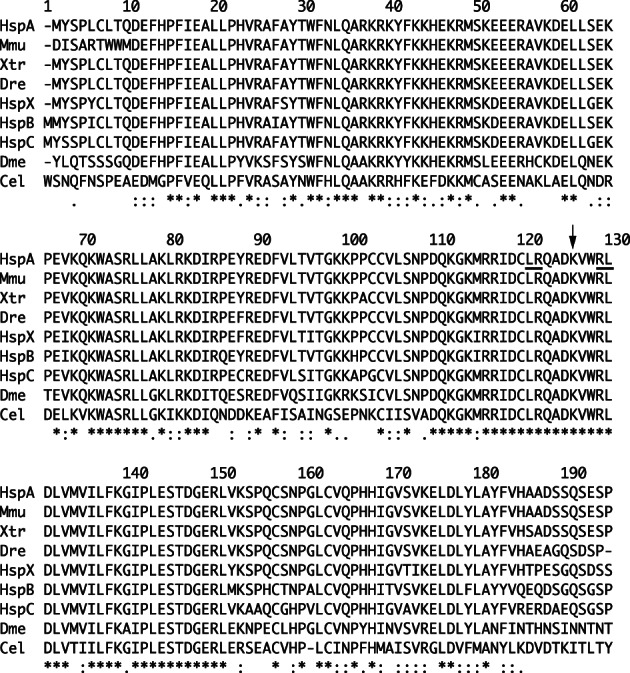
Amino acid sequence alignment of the DNA‐binding domains of human *NFIA* with its orthologs and paralogs. Sequences and domain definitions were obtained from UniProt (The UniProt Consortium, 2019). HspA: human *NFIA* (Q12857, residues 1–194); HspB: human *NFIB* (O00712, 1–195); HspC: human *NFIC* (P08651, 1–195); HspX: human *NFIX* (Q14938, 1–194); Mmu: mouse *Nfia* (Q02780, 24–217); Xtr: frog *nfia* (A0A6I8RQ42, 1–194); Dre: zebrafish *nfia* (F1R2R8, 1–193); Dme: fly *NfI* (Q86P06, 19–212); and Cel: nematode *nfi‐1* (Q09631, 44–237). The K125E mutation site is indicated by an arrow; 6th and 7th mutagenized sites in Armentero et al. (1994) are underlined

Trio exome analysis showed that both patients had the same de novo nonsynonymous mutation in the *NFIA* gene (NM_001134673.4), c.373A>G (p.Lys125Glu). The DNA‐binding domains of *NFIA* and its paralogs are highly conserved and the K125 residue is identical across vertebrates and invertebrates studied (Figure 2). Indeed, the combined annotation‐dependent depletion scores (Kircher et al., 2014) for the K125E variant was 26.8. This variant was absent in the database of 3552 normal Japanese individuals (Japanese Multi Omics Reference Panel:jMorp) (https://jmorp.megabank.tohoku.ac.jp/202001/variants) (Tadaka et al., 2019) and also absent in the Genome Aggregation Database (gnomAD) (http://gnomad.broadinstitute.org/).

### 4.2 Functional assays of K125E variant in *Drosophila*

**FIGURE 3:**
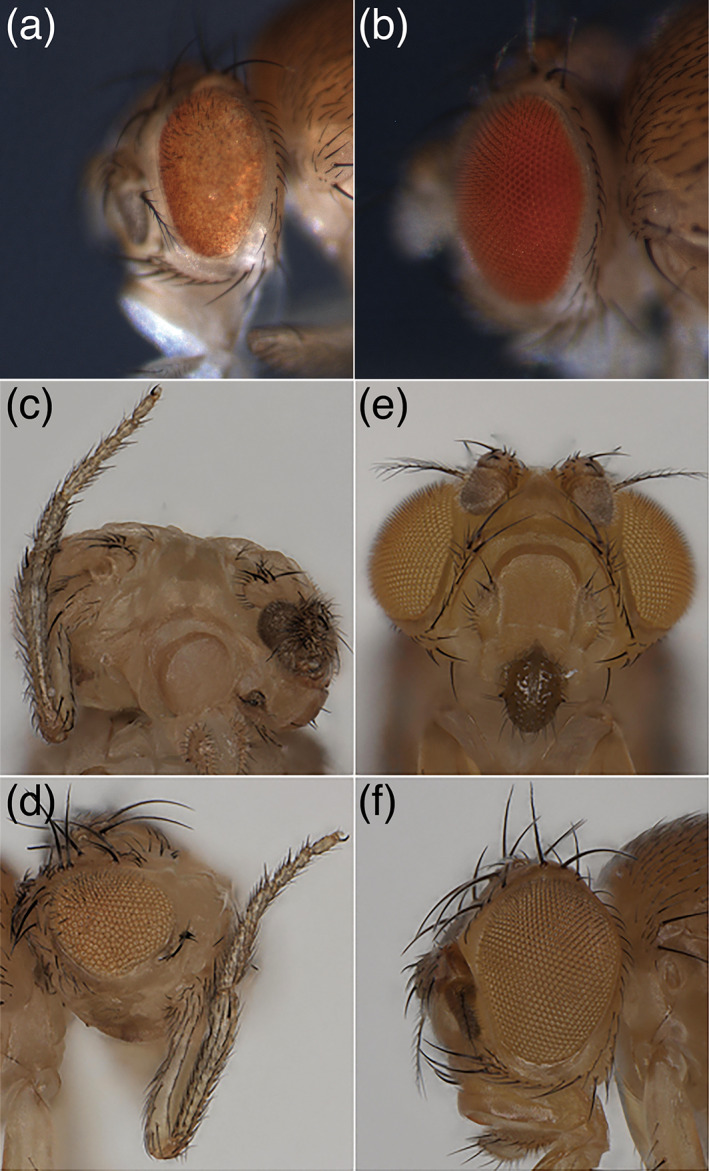
Phenotypes of ectopic expression of *NFIA* gene in *Drosophila*. Ectopic expression of *NFIA* ^*WT*^ (a) but not *NFIA* ^*K125E*^ (b) during retinal development (*GMR>NFIA* ^*WT*^) caused severe neurodegeneration (rough eye phenotype). Ectopic expression of *NFIA* ^*WT*^ (c and d) but not *NFIA* ^*K125E*^ (e and f) under the control of the *ey‐GAL4* driver caused antenna‐to‐leg transformation. Note that the *ey* gene is expressed in the eye‐antennal disc primordia and ubiquitously in the first instar larval disc (Quiring et al., 1994; Urbach & Technau, 2003) [Color figure can be viewed at wileyonlinelibrary.com]

To assess the functional significance of the K125E mutation in vivo, we introduced the wild‐type and mutant human *NFIA* transgenes into *Drosophila* and expressed them in the nervous system and imaginal discs using the GAL4/UAS system. We found that ectopic expression of wild‐type *NFIA* allele (*NFIA* ^*WT*^) in the *Drosophila* retina by the *GMR‐GAL4* driver caused defects in eye development (Figure 3a). However, the K125E allele (*NFIA* ^*K125E*^) did not cause any change in the morphology (Figure 3b). Likewise, when driven by the pan‐neuronal *nSyb‐GAL4* driver, *NFIA* ^*WT*^, but not *NFIA* ^*K125E*^, caused embryonic or first larval lethality (data not shown). What is more, *NFIA* expression by the *ey‐GAL4* caused an antenna‐to‐leg transformation (Figure 3c,d). The well‐known antenna‐to‐leg transformation by ectopic expression of the HOM‐C genes (e.g., *Antennapedia*) is due to suppression of *homothorax* (*hth*) gene transcription and subsequent failure of nuclear localization of *Extradenticle* homeodomain protein (Yao et al., 1999). We then searched the sequence of the fly *hth* gene (*Drosophila melanogaster* R6.37; Larkin et al., 2021) and identified a consensus binding site for *NFIA* (5′‐TTGGCNNNNNGCCAA‐3′, although the two half sites are separated by four instead five nucleotides) approximately 450 bp upstream of the transcription start site. It is likely that *hth* gene transcription was suppressed by ectopic expression of *NFIA* in the first instar larvae when the eye and antenna fates are not yet segregated (Wang & Sun, 2012). By contrast, expression of *NFIA* ^*K125E*^ by *ey‐GAL4* did not have any effect either (Figure 3e,f). In sum, all of the dominant effects of *NFIA* expression were completely abolished in the mutant allele.

### 4.3 Functional assays of K125E variant in zebrafish

**FIGURE 4:**
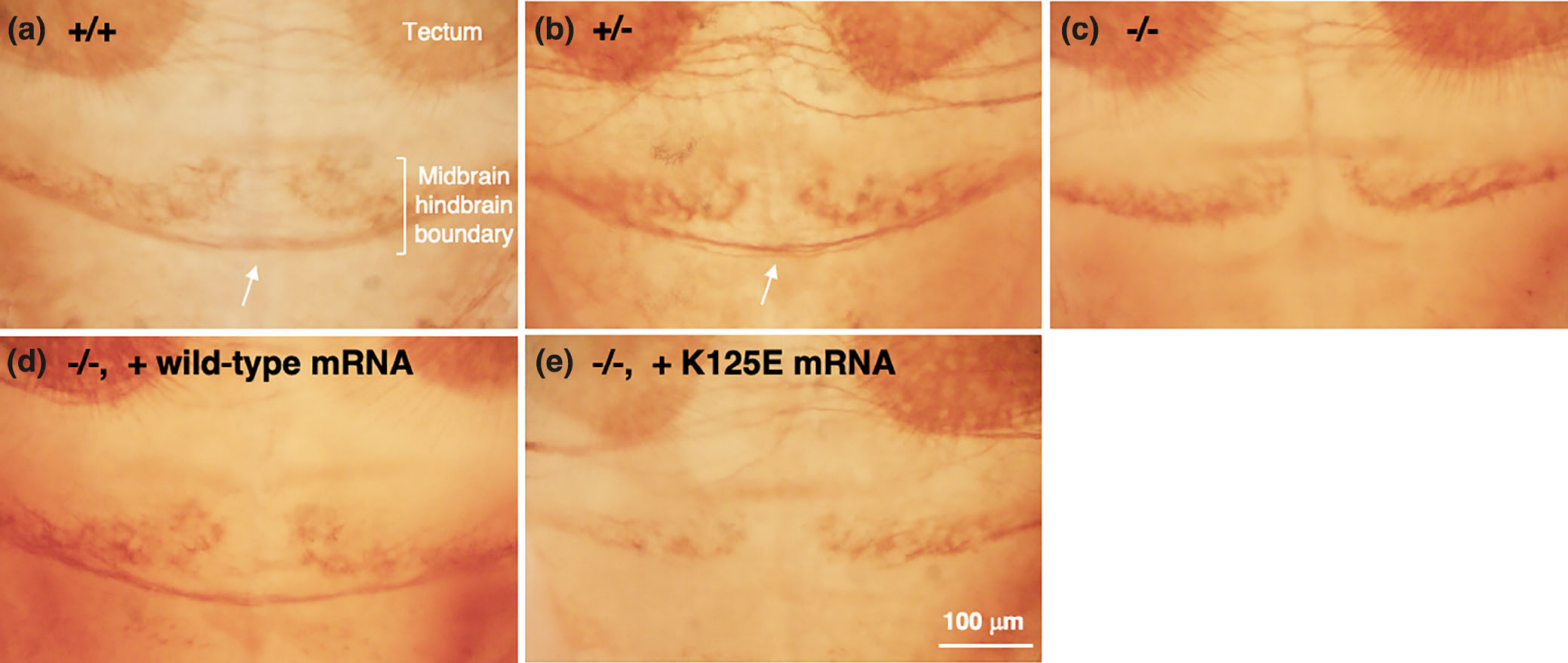
Commissural defects in *nfia* mutant zebrafish. Commissural axons (arrows) cross the midline in the midbrain/hindbrain boundary in wild‐type (a) (*n* = 4/4) and *nfia* heterozygous mutant (b) (*n* = 6/6) embryos at 3 dpf, but many of them failed to do so in *nfia* homozygous mutants (c) (*n* = 5/5). The midline crossing defects in *nfia* homozygous mutants were rescued by injection of *NFIA* ^*WT*^ mRNA (d) (*n* = 5/5), but not by that of *NFIA* ^*K125E*^ one (e) (*n* = 5/5). Axons were labeled with anti‐acetylated α‐tubulin antibody [Color figure can be viewed at wileyonlinelibrary.com]

To further address the functional consequences of the K125E mutation in a vertebrate model, zebrafish, we employed the *nfia* nonsense mutant (*nfia* ^*sa16768*^) as an *NFIA*‐deficient animal model and performed an mRNA rescue assay. As described previously (Chitnis & Kuwada, 1990; Wilson et al., 1990), commissural axons crossed the midline in the midbrain/hindbrain boundary region in wild‐type and *nfia* heterozygous mutant embryos at 3 dpf (Figure 4a,b), but the axons did not cross the midline in *nfia* homozygous mutant (Figure 4c). The lack of commissural fibers in mutants was rescued by injection of wild‐type human *NFIA* ^*WT*^ mRNA (Figure 4d). On the other hand, the injection of *NFIA* ^*K125E*^ mRNA failed to rescue the phenotype (Figure 4e). Collectively, these zebrafish assays indicate that *nfia* is essential for neural development and suggest that *NFIA* ^*K125E*^ is a loss‐of‐function mutation of human *NFIA*.

### 4.4 K125E mutation impaired the transcriptional regulation ability of NFIA

**FIGURE 5:**
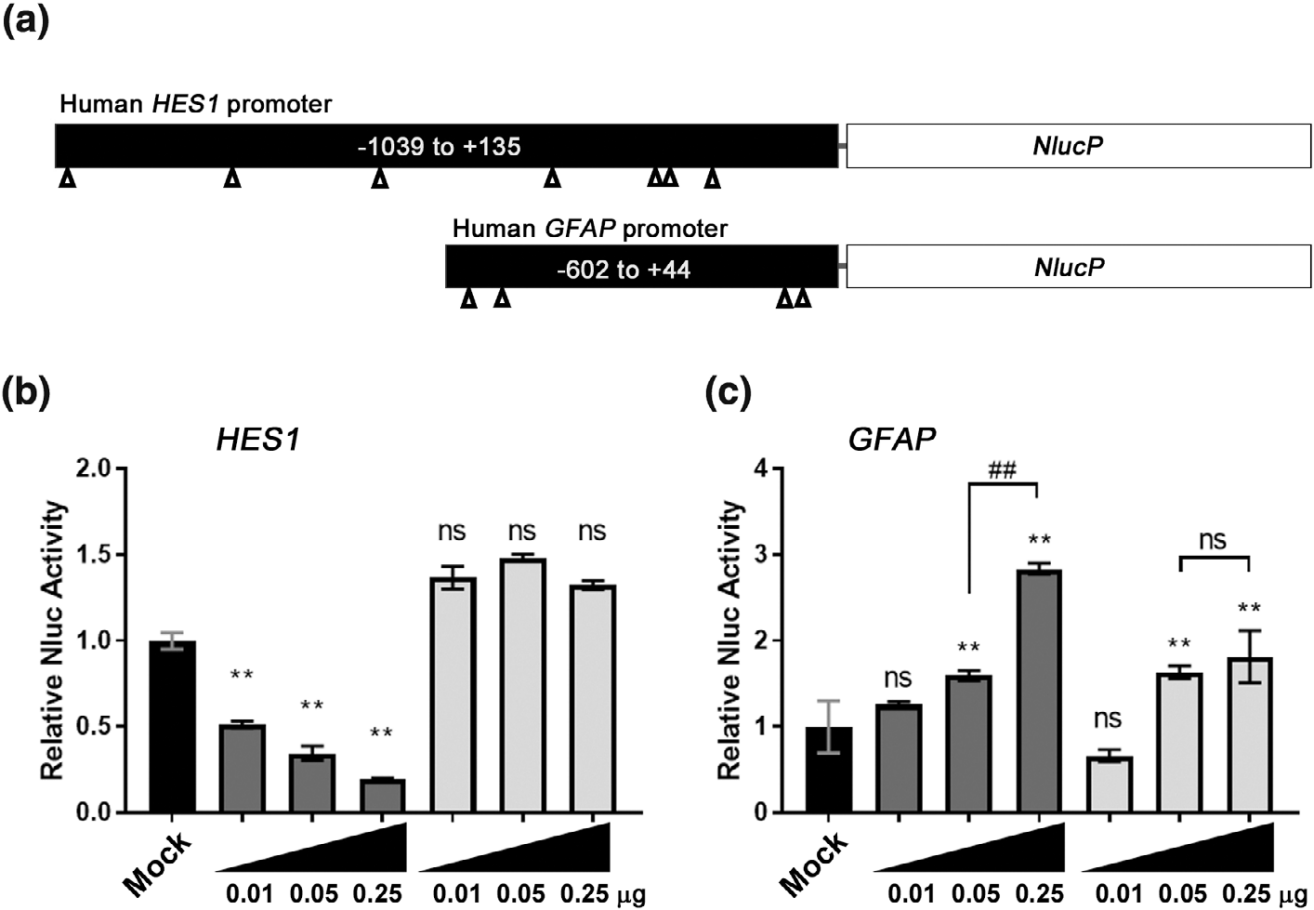
*HES1* and *GFAP* promoter assay in HEK293T cells. (a) Schematic representation of the human *HES1* promoter‐NlucP and the human *GFAP* promoter‐NlucP constructs. Potential NFI‐binding sites (NNTTGGCNNNNNNCCNNN) predicted by TFBIND (http://tfbind.hgc.jp/; Tsunoda & Takagi, 1999) are shown by triangles. (b) *HES1* promoter repression by NFIA. Dose‐dependent repression of human *HES1* promoter by NFIA^WT^ was not observed in NFIA^K125E^. Data are mean ± *SD* from triplicate experiments. **p < 0.01 by lower‐tailed Dunnett multiple comparisons test (α = 0.05). ns, not significant. (c) *GFAP* promoter activation by NFIA. Dose‐dependent activation of human *GFAP* promoter by NFIA^WT^ was weakened in NFIA^K125E^. Data are mean ± *SD* from triplicate experiments. **p < 0.01 by upper‐tailed Dunnett multiple comparisons test (α = 0.05). ##*p* < 0.01 by unpaired *t*‐test. ns, not significant

Because NFIA is known to repress transcription of *Hes1* and to activate that of *Gfap* by direct binding to the promoter regions in mouse (Miura et al., 1990; Piper et al., 2010), we performed luciferase reporter assay using the human *GFAP* and the *HES1* promoters to determine whether *NFIA* ^*K125E*^ has the ability to regulate transcription (Figure 5a). Induction of exogenous *NFIA* ^*WT*^ expression repressed *HES1* promoter activity in a dose‐dependent manner; however, *NFIA* ^*K125E*^ showed no repressive activity at all in HEK293T cells (Figure 5b). Furthermore, dose‐dependent *GFAP* promoter activation by *NFIA* ^*WT*^ was, although not completely, attenuated in *NFIA* ^*K125E*^ (Figure 5c). These results indicate that the K125E mutation severely impaired the *NFIA* transcriptional activity.

## 5 DISCUSSION

In this article, we report the recurrent heterozygous missense mutation K125E in the *NFIA* gene in two unrelated patients with an intellectual disability, corpus callosum anomaly, and macrocephaly. Our in vitro and in vivo analyses consistently indicate that this variant represents a loss‐of‐function allele. The previous studies proposing haploinsufficiency of the *NFIA* gene as a primary cause of the *NFIA*‐related disorder have relied on deletions or truncating mutations (i.e., frameshift mutation or nonsense mutation) (Bayat et al., 2017; Chen et al., 2011; Hollenbeck et al., 2017; Koehler et al., 2010; Lu et al., 2007; Mikhail et al., 2011; Negishi et al., 2015; Nyboe et al., 2015; Rao et al., 2014; Revah‐Politi et al., 2017; Zhang et al., 2020). A few missense mutations have also been reported to be pathogenic or likely pathogenic (Zenker et al., 2019), but their pathogenicity has not been experimentally verified yet. Therefore, this is the first case report of *NFIA* missense variant associated with the neurodevelopmental disorder.

Nuclear factor I family proteins are found to bind the palindromic consensus sequence as homo‐ or heterodimers (Gronostajski, 2000). Although dimerization is essential for DNA binding, these two activities can be separated by mutations (Armentero et al., 1994). K125 residue is sandwiched between two mutants, 6th and 7th mutations in Armentero et al. (1994); Figure 2), that disrupt the DNA‐binding activity, but the former does not impair the dimerization activity. If the K125E mutant protein can still dimerizes, but cannot bind the target sequences, its detrimental effect may be even stronger than that of truncating variants.

As in Figure 2, the K125 residue is conserved in all four human NFI family genes and the missense mutations in NFIB and NFIX are also classified as pathogenic or probably pathogenic in the ClinVar database (Landrum et al., 2018). In particular, *NFIB* K126E mutation (at the site corresponding to K125 residue in *NFIA*) causes a severe loss of transcriptional activity and is one of the variants associated with intellectual disability and macrocephaly (Schanze et al., 2018). In addition, two mutations at the same K125 residue have been reported in *NFIX*. One is K125E in a patient with Malan syndrome (Gurrieri et al., 2015) and another is K125N in a patient with developmental disabilities (Lu et al., 2017); both patients had macrocephaly. Taken together, all these findings underscore the importance of the K125 residue for NFI function.

The hypoplasia of corpus callosum and macrocephaly may represent diagnostic clues to the *NFIA*‐related disorder. Indeed, macrocephaly was shown in 14 of 14 reported patients with truncating variants or intragenic deletions in the *NFIA* gene (Bayat et al., 2017; Mikhail et al., 2011; Negishi et al., 2015; Nyboe et al., 2015; Rao et al., 2014; Revah‐Politi et al., 2017; Zhang et al., 2020) (Table 1). Consistent with this shared feature, knockout mice for *Nfia*, *Nfib*, and *Nfix* all exhibit severe brain malformations including megalencephaly (Campbell et al., 2008; Chang et al., 2013; das Neves et al., 1999). This megalencephaly is hypothesized to be due to delayed radial glia differentiation, which promotes extended self‐renewal and results in an increased number of neural progenitors (Zenker et al., 2019). Corpus callosum hypoplasia was also shown in 13 of these 14 patients (Table 1) and is indeed an important feature of the *NFIA*‐related disorder. In mouse, formation of the corpus callosum requires astroglial‐mediated remodeling of the interhemispheric midline (das Neves et al., 1999; Gobius et al., 2016). Knockout of *Nfia* and *Nfib* delays differentiation of midline zipper glia cells from radial glia, which prevents normal interhemispheric remodeling and affects subsequent callosal tract formation (Gobius et al., 2016). Our in vitro experiments clearly show that the repressive effect of NFIA on the *HES1* promoter was severely impaired by the K125E mutation (Figure 5b). Therefore, it is possible that cellular differentiation from radial glia was delayed and overgrowth of progenitor cells caused the subsequent macrocephaly in the present patients (Piper et al., 2010). The observation of commissural defects in our zebrafish model deficient for *nfia* also substantiates the association of *NFIA* disruption with hypoplasia of corpus callosum.

**TABLE 1:** Summary of the patients with neurodevelopmental disorder and heterozygous variants in *NFIA*

	Patient 1	Patient 2	Patients with truncating variants or intragenic deletion of the *NFIA* gene	
Variants in *NFIA*	p.Lys125Glu	p.Lys125Glu	p.Gln54ProfsTer49 p.Arg69Ter x3 (two patients were a same family) p.Pro365HisfsTer32 p.Arg74Ter intragenic deletion x8 (each two patients and four patients were same families)	8 patients with truncating variants 8 patients with intragenic deletion
Sex	Female	Male	8 males and 6 females	9 males and 7 females
Developmental delay/intellectual disability	Severe (DQ23)	Mild – moderate	12/14	14/16
Macrocephaly	Present	Present	14/14	16/16
Head MRI	Thin corpus callosum, cyst of septi pellucidi, ventricular wall irregularity, decreased white matter volume	Polycerebral gyrus at parasylvius fissures, cortical dysplasia of bilateral cerebral hemisphere, partial myelination delay, hypoplasia of corpus callosum	Abnormality of corpus callosum: 13/14	Abnormality of corpus callosum: 15/16
Facial dysmorphology	High forehead, small eyes, anteverted nares, depressed nasal bridge, broad columella, thin upper‐lip vermilion, high‐arched palate	High forehead, thick eyebrow, short nose, anteverted nares, long philtrum, thin upper‐lip vermilion, retrognathia	High forehead (8/13), eye anomalies (2/13)	High forehead (10/15) eye anomalies (3/15) (one was not available)
Hearing impairment	Present	Present	2/12	4/14 (two were not available)
Urogenital problems	Absent	Absent	4/13	4/15 (one was not available)

Finally, we anticipate that other *NFIA* missense variants may also be associated with the neurodevelopmental disease. If so, our in vitro and in vivo assays would be a valuable tool for diagnosis, especially for evaluating whether a missense mutation is a loss‐of‐function.

## CONFLICT OF INTEREST

The authors declared no potential conflicts of interest.

## AUTHOR CONTRIBUTIONS

U.T., R.S., Y.O., H.H., K.K., and T.T‐S. wrote the main manuscript text. R.S., Y.O., H.H., K.M., and T.T‐S. designed and performed all the experiments. U.T., A.Y., T.Y., T.F., H.Y., M.Y., H.S., T.T., and K.K. have contributed to data collection and interpretation, and critically reviewed the manuscript. All authors contributed to analysis and interpretation of data. All authors agree to be accountable for all aspects of the work in ensuring that questions related to the accuracy or integrity of any part of the work are appropriately investigated and resolved.

## Data Availability

The data that support the findings of this study are openly available in Database of Pathogenic Variants at https://dpv.cmg.med.keio.ac.jp/dpv‐pub/variants, reference number [DPVS:13139.1].
